# Comparative Assessment of Genetic and Morphological Variation at an Extensive Hybrid Zone between Two Wild Cats in Southern Brazil

**DOI:** 10.1371/journal.pone.0108469

**Published:** 2014-09-24

**Authors:** Tatiane C. Trigo, Flávia P. Tirelli, Thales R. O. de Freitas, Eduardo Eizirik

**Affiliations:** 1 Departamento de Genética, Instituto de Biociências, Universidade Federal do Rio Grande do Sul, Porto Alegre, Brazil; 2 Laboratório de Biologia Genômica e Molecular, Faculdade de Biociências, Pontifícia Universidade Católica do Rio Grande do Sul, Porto Alegre, Brazil; 3 Instituto Pró-Carnívoros, Atibaia, Brazil; BiK-F Biodiversity and Climate Research Center, Germany

## Abstract

Increased attention towards the Neotropical cats *Leopardus guttulus* and *L. geoffroyi* was prompted after genetic studies identified the occurrence of extensive hybridization between them at their geographic contact zone in southern Brazil. This is a region where two biomes intersect, each of which is associated with one of the hybridizing species (Atlantic Forest with *L. guttulus* and Pampas with *L. geoffroyi*). In this study, we conducted in-depth analyses of multiple molecular markers aiming to characterize the magnitude and spatial structure of this hybrid zone. We also performed a morphological assessment of these species, aiming to test their phenotypic differentiation at the contact zone, as well as the correlation between morphological features and the admixture status of the individuals. We found strong evidence for extensive and complex hybridization, with at least 40% of the individuals sampled in Rio Grande do Sul state (southernmost Brazil) identified as hybrids resulting from post-F1 generations. Despite such a high level of hybridization, samples collected in this state still comprised two recognizable clusters (genetically and morphologically). Genetically pure individuals were sampled mainly in regions farther from the contact zone, while hybrids concentrated in a central region (exactly at the interface between the two biomes). The morphological data set also revealed a strong spatial structure, which was correlated with the molecular results but displayed an even more marked separation between the clusters. Hybrids often did not present intermediate body sizes and could not be clearly distinguished morphologically from the parental forms. This observation suggests that some selective pressure may be acting on the hybrids, limiting their dispersal away from the hybrid zone and perhaps favoring genomic combinations that maintain adaptive phenotypic features of one or the other parental species.

## Introduction

Hybridization between species is currently considered to be a natural process that often plays an important role in the evolution of various organisms [1]–[4]. Inter-specific hybridization may have different evolutionary consequences. At one end of the continuum, hybridization may produce only sterile F1s, with the main effect for the involved species being the waste of reproductive effort. On the other end, hybridization may produce fertile F1s that are able to cross with each other and also with the parental species, leading to widespread introgression that may induce complete admixture between the two original organisms [5], [6]. The characterization of the nature of each particular hybrid zone, with the identification of its history and the main forces promoting its formation and maintenance, is crucial because these aspects may lead to relevant considerations of the management and conservation of the species involved [7].

An initial and important issue in the investigation of a hybrid zone is the accurate identification of hybrids and parental types. The detection of hybrid individuals relied upon morphological characteristics until the mid-1960s. However, the use of morphological features alone to distinguish between pure and admixed individuals is often inappropriate, because not all morphological variation has a genetic basis, and most of the phenotypic characters have multifactorial determinants [7]. Additionally, morphological characters do not allow one to determine whether an individual is a first or later generation hybrid, which is crucial to the accurate characterization of hybrid zones. The development of new molecular techniques and powerful statistical tools for individual-based analysis [8], [9] allows for the more precise identification of hybrids, as well as the proportion of admixture at the individual or population levels [10], [11], [12]. These pieces of information greatly contribute to shedding light on important aspects of hybrid-zone formation and evolution, including the magnitude, symmetry and consequences of genetic introgression.

Hybridization between two endangered Neotropical small cats, *L. geoffroyi* and *L. guttulus* (recently recognized as a distinct species from *L. tigrinus* [13]), was first documented with the analysis of microsatellites and mitochondrial DNA (mtDNA) sequences [14]. These two species present basically parapatric geographical distributions in the Neotropical Region, with *L. guttulus* occurring mostly in the Atlantic Forest biome of southern and southeastern Brazil, Paraguay and probably northern Argentina, while *L. geoffroyi* ranges mostly through the pampas biome of southern South America [15]–[17]. The overlap between their distributions seems to be quite limited, with an extremely restricted contact zone having been documented in the southernmost Brazilian state of Rio Grande do Sul (RS) [18], [19]. This region was where the first genetic study indicated the occurrence of hybridization between these two species [14]. Subsequently, this inter-specific hybridization was characterized in more detail in a study employing a broader suite of molecular makers, including autosomal microsatellites, mtDNA and nuclear introns located on the X and Y chromosomes [13]. Despite these initial contributions to the understanding of the *L. guttulus vs. L. geoffroyi* hybrid zone, no statistical treatment has so far been applied to verify the predominant genetic categories of hybrids (*e.g.* F1, F2 or backcrosses) in this admixed population, as well to assess the magnitude of hybridization at a local level.

Although previous studies mentioned the existence of some individuals with ambiguous phenotypic characteristics, the great majority of the analyzed animals could be identified to species level on the basis of their morphology. The distinction between the two species was normally based on body size and pelage patterns [15], [17]. In general, *L. guttulus* has a more gracile appearance, with a mean total length of 78.5 cm and mean weight of 2.37 kg, while *L. geoffroyi* is larger and more robust, with a total length and weight varying from 69 to 125 cm and from 2.2 to 7.8 kg, respectively [13], [17]. The *L. guttulus* pelage has a yellowish/ochre background with mostly open rosettes, while *L. geoffroyi* presents a gray/yellowish background pelage color with solid black spots instead of rosettes. In spite of these usual standards for identification, some animals with atypical pelage color, which appeared to be intermediate between the two species, have been documented in RS state since the early 1990s [18], [20]. Although the occurrence of hybridization in that region has now been demonstrated with genetic data, so far no analysis has been performed to test the possible correlation between phenotypes and the genetic status of different individuals.

To better understand the role that hybridization plays in *L. guttulus* and *L. geoffroyi* populations, it is critical to determine the extent and nature of the admixture events occurring between these cats. The genetic study reported in [13] defined several molecular markers that are informative for the investigation of hybridization and introgression between these species, allowing the extent and character of the admixture to be explored in detail. In the present study, we used available genetic data to address the following questions: 1) How extensive is the process of hybridization and introgression in the RS contact zone? 2) What are the predominant hybrid categories occurring in this hybrid zone? 3) Is there evidence of fertility of the hybrids? 4) Can *L. guttulus* and *L. geoffroyi* still be recognized as genetically distinct units in this region despite the observed hybridization? and 5) What is the spatial distribution of hybrids in RS state? In addition to these genetic assessments, we also performed morphometric analyses with a subset of the individuals from the contact zone, aiming to address another set of questions: 6) Can the two species still be confidently separated into distinct morphological groups even in the face of possible extensive hybridization? 7) Is the pattern of morphological variation in this area correlated with the genetic evidence of admixture? and finally 8) Is there evidence of selective pressures acting on this hybrid zone?

## Materials and Methods

### Ethics statement

All biological samples used in this study were previously reported in ref. [13], and were obtained from captive animals or individuals that had been road-killed or caught by farmers. The original collection of biological samples did not involve any capture of live animals, and no collection was performed inside protected or private lands; therefore, there was no requirement of specific permits according to Brazilian law. In addition, no animals were killed or sacrificed for the purpose of this study. Collection of samples was opportunistic and cumulative, *i.e.* it was accomplished over several years via sporadic collection as well as donation of specimens by collaborating research groups. For all the sampled captive animals (*i.e.* zoo animals), all participating zoos provided consent for use of these individuals in the studies. These samples were collected by the zoo staff following their approved procedures (in all cases including appropriate anesthetic protocols). Animals listed as ‘caught by farmers’ comprise individuals that were captured or killed in agricultural areas due to livestock predation conflicts with local landowners, and subsequently confiscated by environmental law enforcement agencies. All such animals included in our study were directly donated to our research group by the responsible environmental agency (federal or state) in charge of the case. Animals that were already dead (sacrificed illegally by farmers) were donated to research (including genetic and morphological analyses reported here). Live confiscated animals were released into the wild by the government agency in charge of the case after collection of blood samples that were donated to our research group. The focal species are considered threatened taxa, but such listing is not an impediment to research. On the contrary, their status as threatened makes them a priority for research, so that Brazilian wildlife authorities support studies on their biology, ecology and genetics that provide critical information for conservation planning on their behalf.

### Features of the genetic data set

**Figure 1:**
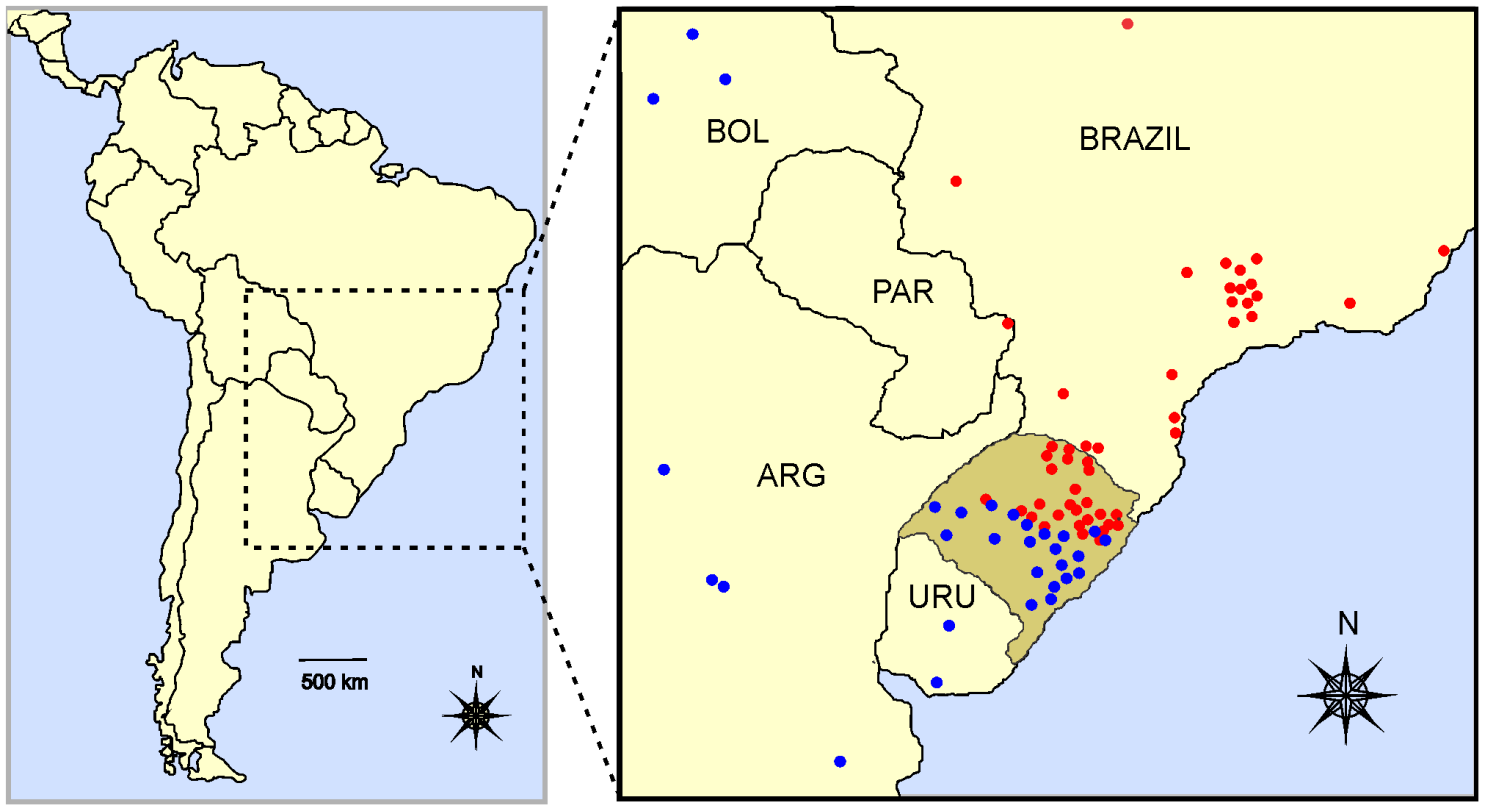
Map showing the location of the study area within South America (left), and an enlarged view (right) depicting sample collection points. Red and blue circles represent collection locales for *Leopardus guttulus* and *L. geoffroyi*, respectively. The area depicted in darker shading in the enlarged map is Rio Grande do Sul (RS) state, where hybridization between these species has been documented. Samples collected inside RS where included in the analyses as the hybridizing populations, while those sampled outside RS were putatively considered to comprise parental/control populations, pending on genetic assessments (see text for details).

The genetic data included in this study was initially reported by [13], and comprised 94 *L. guttulus* and 74 *L. geoffroyi* individuals (Table S1). Of these animals, 45 *L. guttulus* and 49 *L. geoffroyi* were from Rio Grande do Sul state (RS), in southernmost Brazil (Figure 1), where hybridization between these species has been detected. The remaining individuals (49 *L. guttulus* and 25 *L. geoffroyi*) originated from areas farther away from their contact zone (where only one of the two species occurs), and were used here as control/parental populations (Figure 1). These samples were analyzed for a comprehensive suite of molecular markers, including the mtDNA *ND5* gene, two X-linked introns (*PLP1* and *BTK*), two Y-linked introns (*ZFY* and *SMCY3*) and 10 autosomal microsatellite loci (see reference [13] for more details on the molecular markers and data collection).

The identification of each individual was based on external morphology, mainly according the pelage pattern, with individuals presenting open rosettes identified as *L. guttulus* and individuals with solid spots classified as *L. geoffroyi*. However, some specimens collected for this study in RS presented atypical patterns of pelage coloration that could be seen as intermediate between the two species. For these, our morphological identification was based mainly on body size, with the most robust individuals identified as *L. geoffroyi* and those with more gracile appearance as *L. guttulus*.

A sub-sample of these animals was also used for morphological analyses (see below). These analyses comprised only features related to body size, because pelage features were found to be highly variable and thus difficult to quantify and to obtain reproducible categorization in replicated assessments. Therefore, we chose not to perform statistical analyses on them, although our qualitative observations do support the presence of intermediate pelage features in the hybridizing populations of RS.

### Genetic characterization of populations

We initially analyzed the microsatellite data from the control/parental populations, comprising 49 *L. guttulus* and 25 *L. geoffroyi* sampled from areas outside RS state, by employing the Bayesian analysis implemented in STRUCTURE 2.3 [8]. The analysis was performed under a model allowing admixture, assuming correlated allele frequencies between groups [21] and using no prior information of phenotypic (*i.e.* species-level) classification. We performed 1,000,000 Markov Chain Monte Carlo (MCMC) iterations after a burn-in period of 500,000 steps. Five independent runs, each of K = 1–5, were conducted and averaged using CLUMPP 1.1.2 [22]. The CLUMPP output was visualized using DISTRUCT 1.1 [23]. The most likely number of clusters was determined using the ΔK method [24]. This analysis was used to identify the purest individuals of each species among the sampled populations. Only individuals with at least 0.95 of their composite genotype assigned to their phenotype-based population were used as control/parental populations, so as to prevent the genetic influence of potential introgression that could be present in these areas. All the samples from control areas were also assessed for the presence of any introgressed haplotype in the sequence-based markers (mtDNA, X and Y chromosome introns), based on the species-specific haplotypes described in [13] (Table S2, Figure S1). After the definition of the two parental/control groups, the number of private alleles for microsatellite loci was assessed for each of them with the software package ARLEQUIN 3.11 [25].

Samples collected in RS state (n = 94) were initially evaluated for microsatellite loci as a single population, *i.e.* ignoring any morphology-based species identification. This sample was tested for deviations from Hardy-Weinberg Equilibrium (HWE) and linkage equilibrium (LE) using the software packages ARLEQUIN and GENEPOP 3.0 [26], [27]. These samples were also analyzed with STRUCTURE using the same parameters described above for the parental population. The goal of these analyses was to verify the existence of genetic heterogeneity in this population, *i.e.* whether discrete clusters could be identified. To visualize patterns of genetic differentiation between the parental populations and with respect to RS specimens, based on the same set of markers, we performed a Factorial Correspondence Analysis (FCA) using GENETIX 4.05 [28].

We also performed some analyses considering the sample obtained in RS in two distinct populations according the morphological identification. In this sample arrangement, we consider the existence of four populations: parental *L. guttulus*, parental *L. geoffroyi*, RS *L. geoffroyi* and RS *L. guttulus*. HWE and LE were also tested for these populations, and the genetic differentiation with both microsatellite and sequence-based data was assessed with an Analysis of Molecular Variance (AMOVA [29]) implemented in ARLEQUIN, using an F*_ST_* analog [30] and R*_ST_* [31] for the microsatellite data, and Ф*_ST_* (based on p-distances) for the sequence-based data sets. The statistical significance of the observed values was tested using 10,000 permutations. The presence of hybridization in RS was also tested with an allele-based estimate of recent gene flow between the two species using BAYESASS [32], consisting of 3,000,000 iterations, 300,000 steps of burn-in, and sampling of data every 2,000 generations.

### Detection of admixture and assessment of the spatial distribution of hybrids

Admixed individuals in the RS sample were initially detected with the microsatellite data set using two different Bayesian clustering methods, implemented in the packages STRUCTURE and NEWHYBRIDS 1.1 [9]. STRUCTURE was used to assign individuals to populations according to the individual coefficient of membership *q*, following the same conditions described above, with the objective of distinguishing between pure and hybrid individuals. NEWHYBRIDS was used to compute the posterior probability (*Q*) that an individual in the sample belonged to each of six genotypic classes: pure I, pure II, F1, F2 (F1×F1), backcross to I and backcross to II. This analysis was performed using the genotypic classes and allele frequency assumptions described in [9], in runs of 1,000,000 sweeps after a burn-in period of 100,000 sweeps.

To determine the range of expected *q* and *Q* values for each genetic category, and to assess the power of our set of microsatellite loci to distinguish between parental and different hybrid categories, the program HYBRIDLAB 1.0 [33] was used to simulate parental and hybrid genotypes based on our original data. The allelic frequencies estimated from the source parental populations defined by the first STRUCTURE analysis were used to simulate the genotypes of 200 individuals of each parental population. From these, 100 genotypes from each of the following hybrid categories were simulated: F1, F2 and backcrosses to each parental species. The real and simulated genotypes were then run separately in STRUCTURE and NEWHYBRIDS with the same conditions described above.

After the microsatellite-based analysis, each of the RS specimens was examined for its sequence-based markers (mtDNA, X and Y chromosome introns). The presence of any incongruence between the phenotype-based identification and at least one of the three segments was considered to be evidence of potential hybrid origin. This sequence-based hybrid identification was then compared to and integrated with the microsatellite-based hybrid identification, with the aim of assessing the number and proportion of hybrids, and consequently, the extent of hybridization in the RS population.

After these genetic analyses, we evaluated the spatial distribution of all the individuals identified as pure or hybrid. The genetic status of each sample was combined with its geographic location, allowing each individual to be plotted on a map to assess the spatial distribution of pure *L. guttulus*, pure *L. geoffroyi* and hybrids.

### Analysis of morphological variation and correlation with genetic and spatial variation

We obtained a sample of 43 road-killed individuals (20 identified as *L. guttulus* and 23 as *L. geoffroyi*) that were suitable for morphometric analysis (*i.e.* with minimum damage and thus permitting the precise collection of the predefined measurements) (Table S1). All the analyzed specimens were from RS state, with the exception of two *L. guttulus* individuals from the adjacent Santa Catarina state, located immediately north of RS. Twenty-five measurements were taken from each specimen, including 22 body dimensions and three tooth measurements: body length, tail length, head length, weight, shoulder height, neck circumference, breast circumference, head circumference, ear length, posterior length foot, fore paw length, fore paw width, hind paw length, hind paw width, fore footpad length, fore footpad width, hind footpad length, hind footpad width, fore toe length, fore toe width, hind toe length, hind toe width, upper canine width, lower canine width, upper fourth pre-molar length.

Some individuals of our sample (13 *L. guttulus*, and 12 *L. geoffroyi*) were independently measured by two different researchers. Student's test (*t*-test) for paired samples was used to assess the existence of significant differences between these paired measures for each species, independently. Only two measurements (ear length and hind toe width) differed significantly between the two independent researchers for both species (p<0.05) and were thus excluded from further analysis, since these data were considered less reliable.

After this preliminary test, only the measurements taken by one of the researchers were analyzed subsequently. To investigate the structure of morphological diversity in our sample we performed a Principal Component Analysis (PCA) over the variance-covariance matrix, using the software SPSS (SPSS Inc., Chicago, IL, USA). All measurements were standardized by the mean and standard deviation before being subjected to the PCA. For this analysis we chose not to include the phenotype-based identification of each individual, aiming to test whether distinct morphological clusters existed, and if so, whether they correlated with the genetic identification of pure and hybrid individuals. We then employed a univariate analysis (*t*-test) to verify the existence of significant differences between the identified groups. We also performed a Multivariate Analysis of Variance (MANOVA), with a subsequent LSD (Least Significant Difference) *post hoc* test to assess significant differences among groups defined by sex and morphology-driven species identification. Finally, to evaluate the classification of each individual defined as pure or hybrid according to the genetic data into different morphological groups, we conducted a Discriminant Function Analysis (DFA) with SPSS.

We also investigated how morphological and genetic variation found in this sample were related to each other and also how they were spatially distributed, using statistical tests of correlation. Specifically, we aimed to assess whether a geographic pattern of morphological and genetic variation can be discerned in this region, corresponding to the two cat species that are traditionally recognized; or whether the genome and phenotype of the sampled individuals were completely mixed due to extensive hybridization and introgression, obliterating any geographic pattern. If the two cat species are still distinguishable in the admixed RS population, we expected that genetically pure *L. guttulus* would predominate in the northern part of the state, while genetically pure *L. geoffroyi* would predominate in the south. Likewise, smaller individuals bearing the *L. guttulus* phenotype should be found in the north, while larger individuals with the *L. geoffroyi* phenotype would occur in the south.

To construct graphs depicting such relationships, as well as to perform correlation analyses, we used the first principal component (PCI) obtained in the PCA as a measure of morphological variation, and the individual coefficient of membership *q* estimated with STRUCTURE as a measure of genetic variation. To evaluate the spatial distribution of these variables, we assessed the geographic distance (in km) of each sample from a line drawn through the central region of RS, lying on parallel 30°S on its eastern side and shifting towards the north on the western end. This geographic limit was defined based on the study of Eizirik et al. [18], which mapped this line as the geographic contact zone between these two cat species in RS. This is the region of intersection between the two biomes associated with each of these species (Pampas with *L. geoffroyi* and Atlantic Forest with *L. guttulus*). Samples collected north (or east) of this line (*i.e.* towards the Atlantic Forest side) received positive values, while those collected south or west (*i.e.* towards the Pampas), received negative values. For each sample, we used the minimum distance (in km) measured in a straight line perpendicular to this central line dividing the two biomes. The PCA scores and the coefficient of membership *q* were standardized to a scale ranging from −2 to +2, while the geographic distances from the central line were log-transformed.

## Results

### Genetic characterization of populations

The first analysis performed with STRUCTURE to verify the genetic composition of the parental/control populations based on the microsatellite data revealed a significant genetic differentiation between *L. guttulus* and *L. geoffroyi*, with a maximum ΔK observed for two populations. Within this sample, 34 *L. guttulus* and 20 *L. geoffroyi* had at least 0.95 assignment of their composite genotype to their sampling population, and were thus included in the respective parental/control group, and representing the purest set of individuals for each species. However, two putatively pure *L. guttulus* presented a signal of introgression in their mtDNA data, and were thus excluded from the control population. Therefore, the final parental data set included 32 *L. guttulus* individuals. Private alleles were detected in both parental populations, totaling 13 in *L. guttulus* and 35 in *L. geoffroyi*. However, when we excluded low-frequency alleles (frequency <0.05), this number declined to eight in the former species and 14 in the latter.

**Figure 2:**
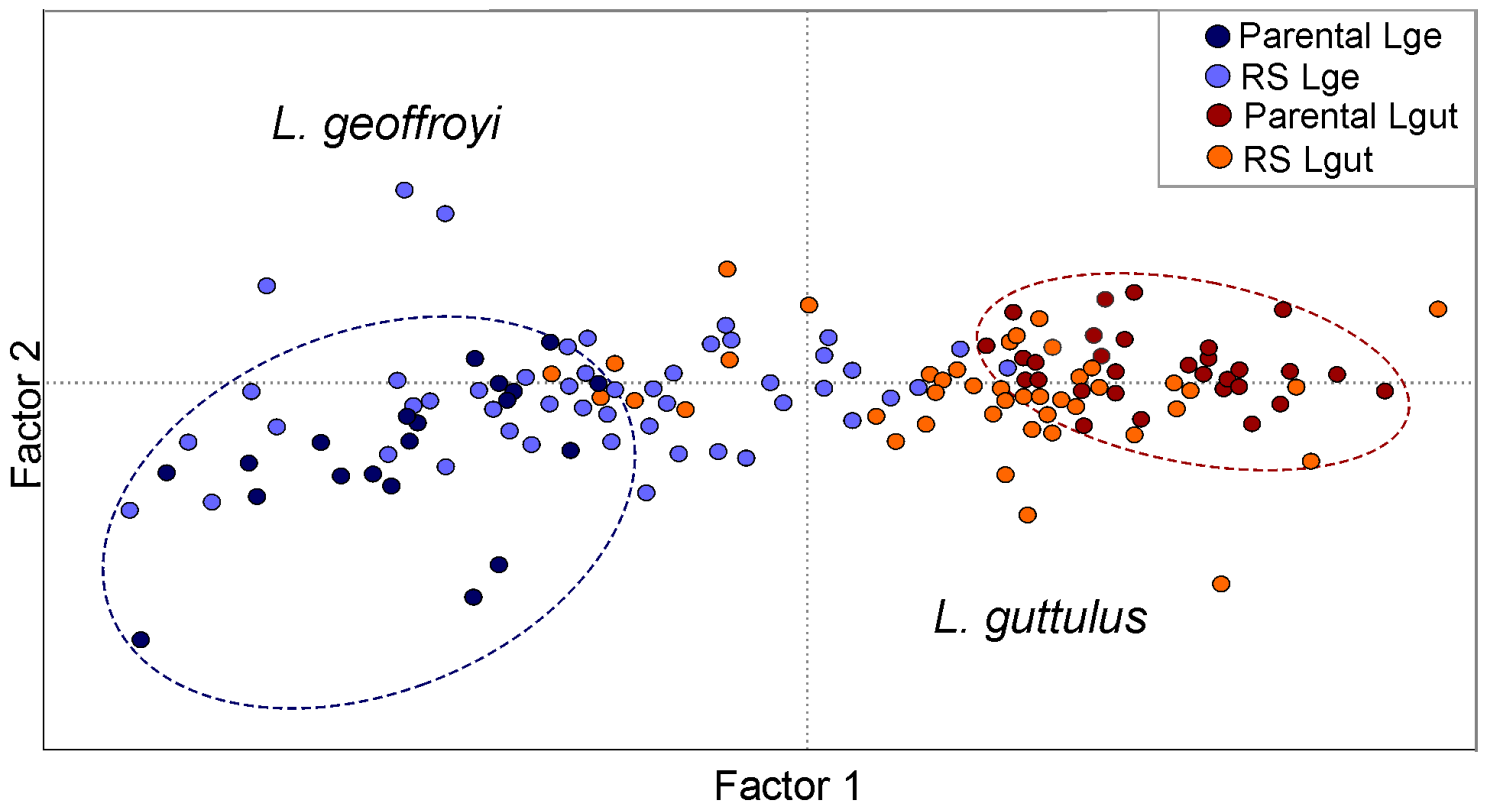
Factorial Correspondence Analysis (FCA) of autosomal microsatellite data. The graph shows the individual scores for parental and hybridizing populations of *Leopardus guttulus* and *L. geoffroyi* on the first two factor axes. Parental populations are delimited by circles (*L. guttulus* – dark red; *L. geoffroyi* – dark blue).

Despite the reduced number of private alleles, genetic differentiation was clear between these two populations, as demonstrated by high and significant values of *F_ST_, R_ST_* and Ф*_ST_* for all molecular markers (microsatellite, mtDNA, X and Y chromosome introns; Table 1). Differences in the results of HWE and LE performed for the two parental populations treated as a single population *vs.* separately also confirmed the existence of genetic distinctiveness between them. While 22 pairwise combinations of loci presented significant deviations from LE when we assumed a single population, only five and two combinations presented significant deviations for parental *L. guttulus* and *L. geoffroyi*, respectively. Likewise, two loci presented significant deviations from HWE when we assumed a single population, but none when we divided the sample into the two parental populations. The FCA based on the microsatellite data corroborated these findings, with the plot showing a marked segregation between the two parental populations (Figure 2).

**Table 1:** Levels of genetic differentiation between parental populations of both species and between samples collected from Rio Grande do Sul state (RS).

	Parental Lgut *vs*. Lge	RS Lgut *vs.* Lge
Microsatellite	0.156/0.324	0.050/0.070
mtDNA	0.930	0.519
X-Chromosome	0.804	0.503
Y-Chromosome	1.000	0.523

Microsatellite data were evaluated with *F_ST_* (left) and *R_ST_* (right). Sequence-based markers, including mitochondrial DNA (mtDNA), X-linked and Y-linked introns, were evaluated with Ф*_ST_*. All values were statistically significant (p<0.001).

Lge  =  *Leopardus geoffroyi*; Lgut  =  *L. guttulus*.

In contrast, when we included the RS samples in the FCA, we observed a clear reduction in this genetic distinction between the two groups. When we considered the *a priori* phenotypic identification of each specimen, we could not distinguish two clear-cut populations, but only a predominance of each species on either side of the plot, with considerable overlap at the center (Figure 2). When we treated the RS sample as a single population (*i.e.* ignoring the phenotype-based species identification), we observed a reduced number of locus combinations deviating from LE (22 in parental populations *vs.* only 10 in RS), but four loci with deviations from HWE. When we assumed the RS sample to comprise two populations (based on the phenotypic identification), these numbers declined to only one and four combinations deviating from LE in *L. guttulus* and *L. geoffroyi*, respectively, and only two loci with deviations from HWE for each population. The existence of substantial deviations from LE and HWE in the RS sample (when treated as a single unit) was reflected in the STRUCTURE analysis, which yielded a maximum ΔK for two populations when this set of individuals was assessed.

The results obtained for the RS sample indicated a reduced genetic distinctiveness between the two cat species in this region when compared to the parental populations, but suggest that some separation still occurs in this area. Standard measures of genetic differentiation between parental populations and between phenotypic groups sampled in RS also reflected this pattern. While some significant genetic differentiation was detected between the RS phenotype-based populations for all the molecular markers we evaluated, these values were *ca.* 50% lower than those obtained between parental populations (Table 1). In addition, estimates of current gene flow between the two RS phenotype-based populations, generated using BAYESASS, were considerably higher than between the parental populations, with migration rates presenting a mean of 0.3210 (standard deviation of 0.012) for the former comparison, in stark contrast to a mean of 0.0098 (standard deviation of 0.009) for the latter.

### Detection of hybrids, quantification of admixture and spatial distribution of hybrids

**Figure 3:**
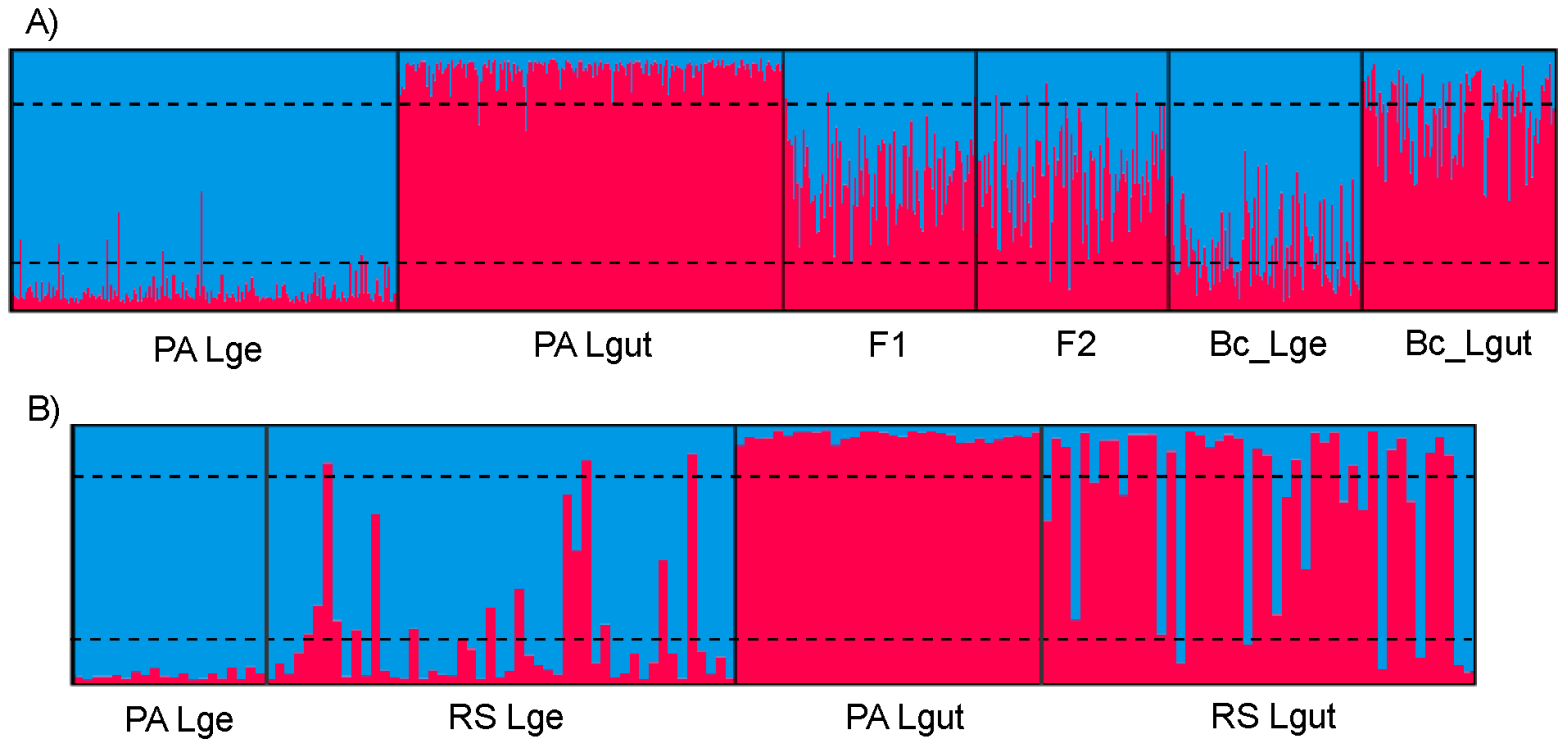
Bar plotting of the results obtained with STRUCTURE using K = 2. Each individual is represented by a vertical bar, with the colors indicating the proportion of its genomic membership in (*i.e.* assignment to) the two clusters: *L. geoffroyi* (blue) and *L. guttulus* (red). A) Simulated genotypes belonging to *Leopardus geoffroyi* and *L. guttulus* parental groups (PA Lge and PA Lgut), and the four hybrid categories: F1, F2, backcross with *L. geoffroyi* (Bc_Lge) and backcross with *L. guttulus* (Bc_Lgut). B) Real data from parental/control *L. geoffroyi* and *L. guttulus* (PA Lge and PA Lgut, respectively) and hybridizing populations from Rio Grande do Sul state (RS Lge and RS Lgut). The two horizontal dashed lines indicate the threshold of 0.8 assignment to each cluster.

The STRUCTURE analysis of the simulated genotypes revealed that the parental populations were assigned to their correct group with an average *q_1_* of 0.934 (0.696–0.974) for *L. guttulus*, and *q_2_* of 0.928 (0.543–0.974) for *L. geoffroyi* (Table 2, Figure 3). Using a threshold *q*-value of 0.9, 88.5% of the parental *L. guttulus* individuals and 83.5% of the *L. geoffroyi* were correctly assigned to their species-level group. These values reached 99% and 96.5%, respectively, when a threshold of 0.8 was used, increasing the efficacy of detection of pure individuals. On the other hand, this lower threshold resulted in a reduced efficacy in the detection of hybrid individuals. Nevertheless, for both of these thresholds, the proportion of F1 or F2 hybrids that was incorrectly identified was at most 9% (Table 2). The categories that were most affected by the different thresholds were the backcrosses, for which we observed a reduction of 30–34% in the efficiency of detection using the 0.8 threshold instead of 0.9. The *q*-values generated from first-generation backcrosses into *L. guttulus* ranged from 0.420 to 0.956, with a mean association of 0.759 to this species-level group; similarly, *L. geoffroyi* backcrosses ranged from 0.381 to 0.967 assignment to this species, with a mean association of 0.768 (Table 2).

**Table 2:** Results of the Bayesian analysis performed with STRUCTURE on the simulated genotypes.

	Average proportion of assignment		Percentage of correct assignment	
	*q1*	*q2*	0.8	0.9
Parental Lge	0.072	0.928	96.5%	83.5%
Parental Lgut	0.934	0.066	99%	88.5%
F1	0.519	0.481	96%	100%
F2	0.520	0.480	91%	99%
Bc_Lge	0.232	0.768	48%	82%
Bc_Lgut	0.759	0.241	55%	85%

The values include the average proportion of assignment of each simulated hybrid category considering two clusters, and the percentage of correct assignment of each category under two thresholds (0.9 and 0.8).

Lge  =  *Leopardus geoffroyi*, Lgut  =  *L. guttulus*, Bc  =  backcross (the parental direction of the backcross is indicated on the right).

With respect to the distinction among different hybrid categories, the analysis of the simulated data with NEWHYBRIDS yielded inconclusive results. A reasonable efficiency in the detection of simulated parental genotypes was only achieved with a threshold of *Q* = 0.6, resulting in 95% correct assignment for *L. guttulus* and 95.5% for *L. geoffroyi*. However, with this threshold the majority of the simulated hybrid genotypes for F1, F2 and backcrosses exhibited similar assignment probabilities to two or more of the four hybrid categories, making it impossible to perform accurate estimates on admixture ancestry. However, when we pooled the association values of each individual to all hybrid categories, 96% and 88% of the simulated F1 and F2, respectively, were defined as a hybrid with *Q*>0.6. On the other hand, even using this approach, the simulated backcrosses showed low rates of correct classification, reaching only 68% for *L. guttulus* and 46% for *L. geoffroyi*, with several simulated hybrid individuals assigned to pure categories under the 0.6 threshold.

The analyses performed with the real data set identified 29–43 likely hybrids in RS state with STRUCTURE (depending on the threshold used: 0.8 or 0.9) and 42 with NEWHYBRIDS (using a threshold of 0.6) (Table 3). In most cases, individuals were clearly identified as hybrids with both programs (*q*<0.8 and *Q*<0.6). However, there were some discrepancies in which one individual was identified as pure with one analysis (*q*>0.8/0.9 or *Q*>0.6) and as hybrid with the other one. In those situations, we only considered conclusively those individuals to be hybrids where there was additional evidence of admixture in the sequence-based markers.

**Table 3:** Absolute number and percentage of identified hybrid and pure individuals in the Rio Grande do Sul (RS) sample under the thresholds established for each of the microsatellite analyses (0.8/0.9 for STRUCTURE, and 0.6 for NEWHYBRIDS).

	STRUCTURE		NEWHYBRIDS	Sequence
	0.8	0.9	0.6	
Pure *L. geoffroyi*	35 (71.42%)	26 (53.06%)	22 (44.90%)	28 (57.14%)
Hybrid *L. geoffroyi*	14 (28.58%)	23 (46.94%)	27 (55.10%)	21 (42.86%)
Hybrid *L. guttulus*	15 (33.33%)	20 (44.44%)	15 (33.33%)	9 (20.00%)
Pure *L. guttulus*	30 (66.67%)	25 (55.55%)	30 (66.67%)	36 (80.00%)

The equivalent results obtained with the sequence-based molecular markers are shown on the far right column.

For both phenotype-based RS populations, some individuals identified as pure with all microsatellite analyses (*i.e.* presenting *q*>0.8/0.9 for STRUCTURE and *Q*>0.6 for NEWHYBRIDS), showed a signal of genetic introgression in at least one of the sequence-based markers (*e.g*. bLge01, bLgut05), and may be considered a result of later F*n* or backcross generations (Table 4). Only two individuals identified morphologically as *L. geoffroyi* (bLge13 and bLge72) showed very low values of association to their original phenotype-based population in the microsatellite analyses, and simultaneously presented all haplotypes assigned to *L. guttulus*.

**Table 4:** Individuals identified as hybrids between *L. guttulus* and *L. geoffroyi* in RS state, Brazil.

*L. geoffroyi*	NH	STR	mtDNA	X	Y	*L. guttulus*	NH	STR	mtDNA	X	Y
bLge01	0.994	0.984	*Lgut*	*Lge*	*Lge*	bLgut01	0.018	0.635	*Lge*	*Lgut*	*
bLge02	0.316	0.924	*Lgut*	*Lgut*	*Lgut*	bLgut05	0.760	0.923	*Lgut*	*Lge*	*Lge*
bLge04	0.036	0.883	*Lge*	*Lgut*	*Lgut*	bLgut09	0.001	0.247	*Lge*	*Lgut*	*
bLge05	0.001	0.807	*Lge*	*Lgut*	*Lge*	bLgut47	0.249	0.676	*Lgut*	*Lgut*	*
bLge06	0.172	0.697	*Lgut*	*NA*	*NA*	bLgut49	0.000	0.054	*Lge*	*Lgut*	*
bLge07	0.000	0.140	*Lgut*	*Lgut*	*Lge*	bLgut68	0.961	0.958	*Lgut*	*Lge*	*Lgut*
bLge08	0.006	0.756	*Lgut*	*Lgut*	*Lge*	bLgut79	0.000	0.098	*Lge*	*Lgut/Lge*	*
bLge10	0.951	0.974	*Lge*	*Lgut*	*	bLgut98	0.000	0.068	*Lgut*	*Lgut*	*
bLge11	0.015	0.792	*Lgut*	*Lge*	*Lge*	bLgut108	0.031	0.734	*Lgut*	*Lgut*	*Lgut*
bLge12	0.963	0. 971	*Lge*	*Lgut*	*Lgut*	bLgut119	0.000	0.191	*Lgut*	*NA*	*Lge*
bLge13	0.000	0.340	*Lgut*	*Lgut*	*Lgut*	bLgut121	0.000	0.078	*Lge*	*Lgut*	*Lgut*
bLge32	0.011	0.787	*Lge*	*Lgut*	*Lge*	bLgut135	0.000	0.158	*Lgut*	*Lgut*	*
bLge42	0.004	0.706	*Lge*	*Lgut*	*Lgut*	bLgut138	0.194	0.268	*Lgut*	*Lgut*	*
bLge46	0.001	0.631	*Lgut*	*Lge*	*Lgut*	bLgut141	0.002	0.443	*Lgut*	*NA*	*Lgut*
bLge49	0.712	0.932	*Lge*	*Lgut*	*Lgut*	bLgut143	0.862	0.943	*Lgut*	*Lgut/Lge*	*
bLge72	0.000	0.265	*Lgut*	*NA*	*Lgut*						
bLge73	0.000	0.483	*Lge*	*Lgut*	*Lge*						
bLge74	0.000	0.131	*Lgut*	*Lge*	*						
bLge76	0.038	0.773	*Lge*	*Lge*	*Lge*						
bLge79	0.071	0.887	*Lgut*	*Lgut*	*Lgut*						
bLge80	0.990	0.985	*Lgut*	*Lge*	*Lgut*						
bLge90	0.036	0.520	*Lge*	*Lge*	*Lgut*						
bLge93	0.000	0.105	*Lgut*	*Lge*	*						

NH and STR are the probabilities of association of each individual to its phenotypic population based on the NEWHYBRIDS and STRUCTURE analyses of microsatellites, respectively. The three columns labeled ‘mtDNA’, ‘X’ and ‘Y’ correspond to the species-specific haplotypes from sequence-based markers (mitochondrial DNA, X and Y chromosome introns, respectively) observed for each specimen.

*female individual, NA  =  not analyzed, *Lge*  =  haplotype inferred to originate from *L. geoffroyi, Lgut*  =  haplotype inferred to originate from *L. guttulus*.

Evaluating all the data sets jointly (microsatellite loci and sequence-based markers), and considering the criterion described above with a threshold of 0.8 for STRUCTURE results, we could observe a large number of complex genetic combinations, with 23 *L. geoffroyi* and 15 *L. guttulus* presenting strong evidence of hybrid ancestry. The observed combinations did not reveal any clear case of F1 hybrids in either phenotype-based population, with the majority being compatible with F2 hybrids and/or backcrosses involving both parental species (Table 4). Interestingly, in spite of the low accuracy observed in the classification of the simulated genotypes into different hybrid categories with NEWHYBRIDS, several identified hybrids in the real data set showed high values (*Q*>0.6) of association to the F2 category, reaching 75% of the hybrids in the RS phenotype-based *L. guttulus* population, and 66.67% in the RS *L. geoffroyi* population.

**Figure 4:**
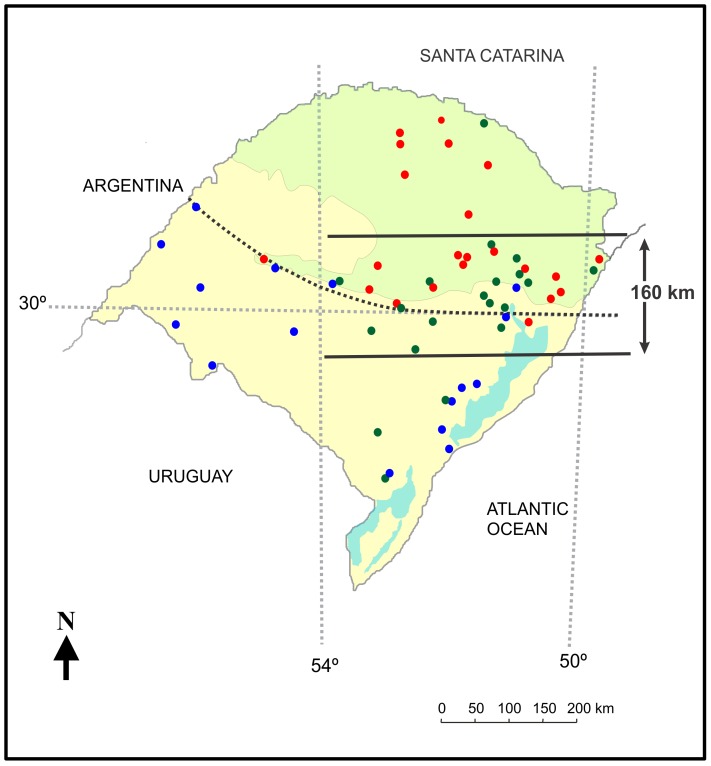
Spatial distribution of samples collected in RS state, including their genetic identification as pure *L. guttulus* (red circles), pure *L. geoffroyi* (blue circles), and hybrids (green circles). Background colors on the map correspond to the two biomes found in RS state: Atlantic Forest (green) and Pampa (tan). The area delimited by the thick lines correspond to a central region spanning *ca.* 160 km in a north-south direction, between longitudes 50°W and 54°W, where the highest concentration of genetically identified hybrids was observed. The dashed black line at the center of the map, lying on parallel 30°S on its eastern side and shifting towards the north on the western end, corresponds to the geographic limit between the distributions of the two cat species [18]. This line was used to evaluate the spatial distribution of genetic and morphological variables (see Figure 6).

The spatial distribution of these identified hybrids was concentrated in the central part of RS state, mostly near parallel 30°S, around the region of intersection between the two biomes found in the state, *i.e.* Pampas in the south and west and Atlantic Forest in the north-east (Figure 4). Most of the hybrids (84%) were sampled in a central region surrounding parallel 30°S and spanning approximately 160 km north-south, between longitudes 50°W and 54°W. We tested whether the proportion of hybrid and pure individuals differed significantly between this central region and the remaining areas of the state, and observed a statistically significant result (χ^2^ = 8.97, d. f. = 1, p<0.01). Individuals identified as pure predominated outside this central region, with *L. guttulus* specimens dominating the northern portion of RS (strongly associated with the Atlantic Forest biome) and *L. geoffroyi* dominating the south and west, in strong association with the Pampas (see Figure 4).

### Morphometric analyses and its correlation with genetic and spatial variation

**Figure 5:**
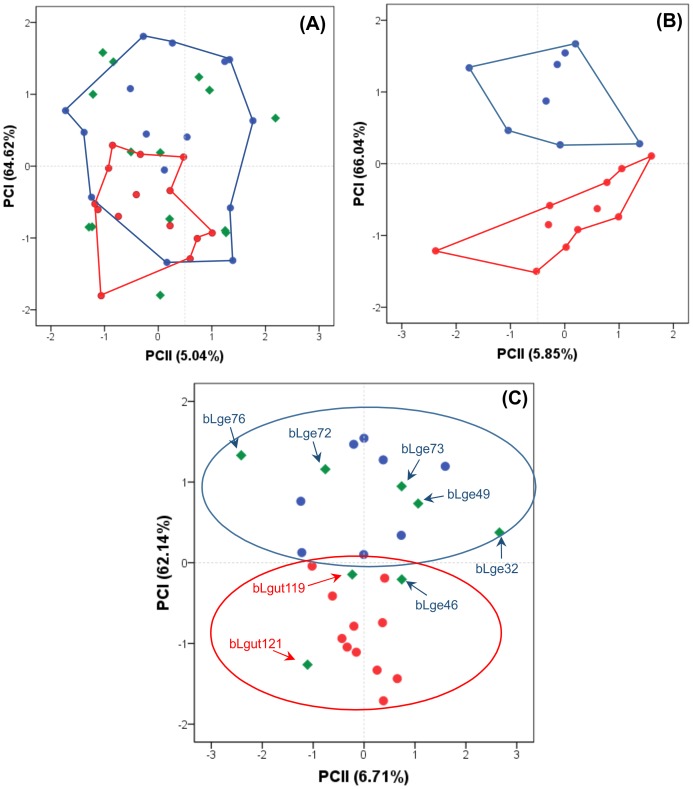
Results of the morphometric analyses. Principal Component Analysis (PCA) results are shown as graphs combining scores of the first (PCI) and second (PCII) principal components for: (A) all the assessed specimens, including information on whether each individual was identified genetically as pure *L. guttulus* (red circles), pure *L. geoffroyi* (blue circles) or a hybrid (green diamonds); (B) only male specimens identified genetically as pure *L. guttulus* (red circles) and pure *L. geoffroyi* (blue circles); and (C) same sample set as in (B), with the addition of genetically identified hybrids. Red and blue polygons (or ellipses) demarcate the morphological space encompassed by pure *L. guttulus* and *L. geoffroyi* individuals, respectively.

The first PCA conducted to explore the morphological variation found in our subset sample from RS and vicinities showed a relatively homogeneous distribution of individuals identified as genetically pure *L. geoffroyi*, pure *L. guttulus* or hybrids (Figure 5A). We could only see a trend for individuals identified genetically as pure *L. guttulus* to occupy the bottom area of the plot, but exhibiting a broad overlap with the *L. geoffroyi* morphological space.

However, a closer inspection of the *L. geoffroyi* samples showing higher overlap with *L. guttulus* in the lower portion of the plot revealed that those individuals were mostly females. Thus, we decided to assess the existence of sexual dimorphism within this species by conducting *t*-tests (which were performed only between males and females identified as pure individuals according to the molecular data). We found that 17 of the 23 measurements evaluated independently showed significant differences between the sexes, indicating the presence of marked sexual dimorphism in *L. geoffroyi*, with males being significantly larger than females. Unfortunately, it was not possible to perform the same analysis for *L. guttulus* due to the small number of females from this species with no evidence of hybridization (n = 2). Nevertheless, we tested the existence of significant differences in body size between samples of *L. geoffroyi* (males and females separately) and *L. guttulus* (both sexes pooled) through a MANOVA. The MANOVA indicated a significant difference among the three groups (F = 43.45, p<0.05), with the LSD *post hoc* test showing significant differences for the majority of the body measurements between male and female *L. geoffroyi*, and between male *L. geoffroyi* and *L. guttulus*, but not between female *L. geoffroyi* and *L. guttulus*.

Given the marked sexual dimorphism detected in *L. geoffroyi* and the size overlap between female *L. geoffroyi* and *L. guttulus*, we decided to conduct the subsequent set of analyses only with the male samples of each species. We initially performed a PCA including only male specimens inferred to be pure (*i.e.* not admixed) with the molecular data. This analysis revealed two clearly distinct groups, with significant differences between them along PCI (*t*-value  = 6.82, d. f. = 17, p<0.001). *L. guttulus* individuals formed a group in the lower area of the plot, and *L. geoffroyi* in the upper area, clearly due to a larger size in the latter species (Figure 5B). The DFA performed with these same samples yielded 100% correct classifications, indicating that the genetic and morphological identification of pure individuals were in complete agreement.

We then included the male specimens identified as hybrids with the molecular data, and repeated the same PCA. The resulting diagram revealed that the majority of the *a priori* species identifications were in agreement with the groups formed by the PCA (Figure 5C). The two hybrids identified *a priori* as more *L. guttulus*-like were positioned near the specimens classified as pure *L. guttulus* based on the genetic data. The same occurred to five of the six hybrid individuals considered *a priori* as more *L. geoffroyi*-like, with the only exception of bLge46, which was positioned near the pure *L. guttulus* samples. The DFA performed with these same samples yielded 92.86% of correct classifications, with only two misclassifications (bLge46 and bLge32). These analyses indicated that most hybrid individuals could be assigned to one or the other morphological population, even in face of the extensive introgression affecting this system.

**Figure 6:**
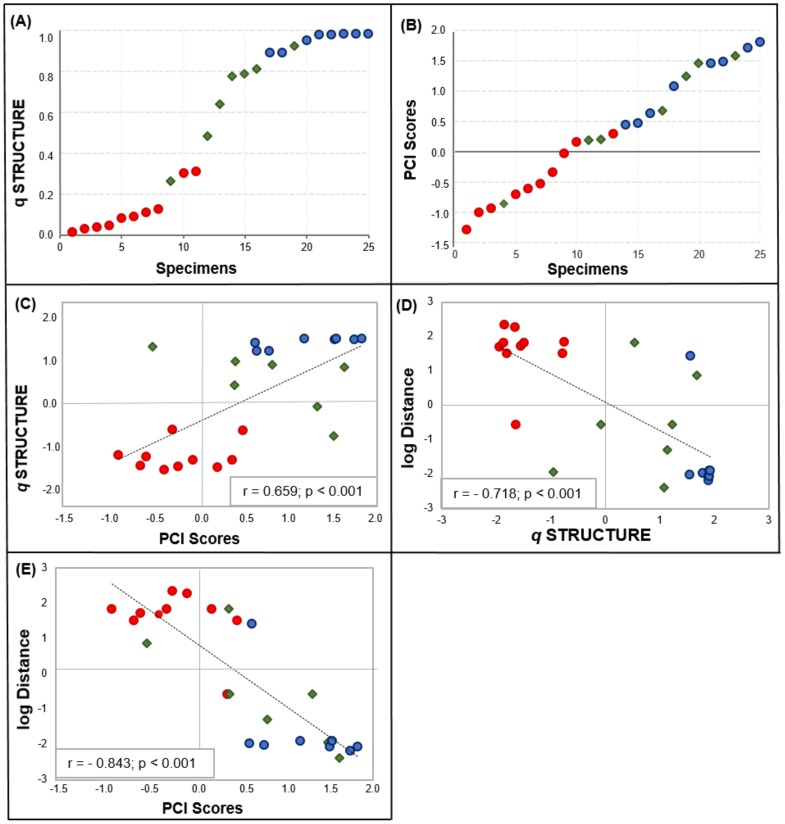
Correlation of morphological, genetic and spatial variables among the male specimens sampled in RS and vicinities. (A–B) patterns of genetic (A) and morphological (B) variation, with individuals ordered on the X-axis based on their ‘*q*’ values estimated with STRUCTURE (in A) or their PCI scores estimated with the PCA (in B); (C) correlation between genetic and morphological variation; (D–E) correlation between individual ‘*q*’ values (D) or PCI scores (E) and spatial transects across the hybrid zone. Geographical distances used in the spatial analyses were measured in km as lines that were perpendicular to the dashed line shown in Figure 4, with samples collected north or east of this line (towards the *L. guttulus* side) receiving a positive value, and those obtained south or west (towards the *L. geoffroyi* side) receiving a negative value - see Methods for more details. Red circles  =  individuals identified as pure *L. guttulus*; blue circles  =  individuals identified as pure *L. geoffroyi*; green diamonds  =  hybrids. PCA scores and the STRUCTURE coefficient of membership *q* were standardized to a scale from −2 to +2, while geographic distances were log-transformed.

Evaluating the patterns of genetic and morphological variation, we observed a correlated gradient between *L. guttulus* and *L. geoffroyi* individuals. For the genetic data, hybrids generally exhibited intermediate *q* values, with exceptions being related to our criterion of hybrid identification (in which intermediate *q* values not corroborated by NEWHYBRIDS or sequence-based markers were not considered definitive hybrids; conversely, individuals with high *q* values but some sequence-based evidence of introgression were considered hybrids) (Figure 6A). Integrating this observed pattern with the morphological variation, we noticed that genetically identified hybrids often did not present intermediate PCI values (Figure 6B). In fact, some hybrids showed almost extreme scores on PCI, indicating that in those cases the molecular evidence of hybridization was not strongly correlated with an intermediate body size. Still, we detected a moderate but significantly positive correlation between the morphological and genetic variation (Figure 6C), possibly driven largely by the pure individuals located on either extreme of the graph (see below).

The morphological and genetic variation found in those male specimens was also evaluated spatially (Figures 6D and 6E). We observed that individuals assigned with both genetic and morphological data to *L. guttulus* were mainly found in the northern portion of RS state, while *L. geoffroyi*-like individuals were mainly in the south, resulting in high and significant (p<0.001) correlation values for both types of data. The correlation with distance from the central zone was higher for the morphological data (r = −0.843) than for the molecular markers (r = −0.718), showing a clear tendency of body size increase towards the southern end of the state (Figure 6E). Interestingly, when we excluded the hybrid individuals from this analysis (not shown), we observed that the re-calculated correlation coefficient was roughly unchanged for the morphology (r = −0.852), but increased substantially for the molecular data (r = −0.857). This result indicates that hybrids add noise to the correlation between geography and genetic data, but behave similarly to the pure individuals when we assess the morphological data.

## Discussion

### Extent and characteristics of the hybridization process in RS state

The genetic analyses performed here have led to an improved understanding of the hybridization process between *L. guttulus* and *L. geoffroyi* in southern Brazil, including an assessment of power and limitations of our present molecular markers. Simulations and assignment tests conducted with the microsatellite data set and the DNA sequence segments allowed the identification of an extensive degree of hybridization, with a significant impact on the genetic composition of both species around their contact zone.

The use of simulated genotypes was extremely useful to assess the power of our ten microsatellite loci to distinguish between pure and hybrid individuals. Through the generation of simulated genotypes, we concluded that the Bayesian analysis performed using STRUCTURE with these markers and the defined parental populations was quite efficient (although not perfect) in distinguishing pure from hybrid individuals under either a 0.9 or a 0.8 threshold. The use of a more conservative threshold (0.8) led to an underestimation of hybrids in the population due to the larger overlap of values found for pure animals and backcrosses. Such a problem has also been reported in other studies that used simulated genotypes in hybrid analyses [10], [11], [34], [35].

The difficulty in distinguishing purebred from backcrosses on the basis of microsatellite data alone was clearly seen in both populations, with *L. guttulus* and *L. geoffroyi* individuals with *q*-values even higher than 0.9 (thus being considered pure) presenting some sequence-based evidence of introgression, implying a hybrid ancestry. In the great majority of hybrid studies, the most complex task is considered to be the identification of backcrossed individuals, especially in the case of later-generation backcrosses [10], [34], [36]. Accordingly, several sources have argued that this distinction often requires a large number of diagnostic alleles and/or a larger number of microsatellite loci [8], [9], [37]. For example, Vähä & Primmer [37], demonstrated that a higher efficiency in distinguishing between purebreds and backcrosses with STRUCTURE was only achieved when as many as 48 microsatellite loci were used, and when the genetic divergence between the hybridizing parental populations was *F*ST  = 0.21.

The assignment of each individual to different hybrid categories using NEWHYBRIDS was largely inconclusive with the simulated data. Therefore, this software was only used in the final analysis as a complement to STRUCTURE in the task of distinguishing pure from hybrid individuals, and for such a purpose it performed well, largely agreeing with the STRUCTURE results. In addition, the inclusion of DNA sequence data from the mtDNA, X-linked and Y-linked segments was important in improving the sensitivity of our hybrid categorization. When we evaluated the entire suite of genetic markers, combining species-specific sequences with microsatellite assignments, we perceived that the identified hybrids were simultaneously compatible with an F2 or backcross status, without a single clear case for a F1 individual. This dominance of post-F1 hybrids with the use of only 10 microsatellite loci without fully diagnostic alleles for each species may underlie the failure of NEWHYBRIDS to distinguish among the different hybrid categories [9].

These findings indicate that hybrids generated from initial crosses between *L. guttulus* and *L. geoffroyi* are viable and at least partially fertile. The fertility of hybrids could be directly documented by the example of one road-killed and pregnant female (bLgut79) identified morphologically as *L. guttulus* and recognized as a hybrid with our molecular data. On the other hand, even the combination of sequences and microsatellite assignments did not allow us to clearly identify the predominant post-F1 category occurring in this system. Therefore, the molecular tools used in this study were still insufficient to accurately determine whether the main crosses occurring in this hybrid zone involve mainly backcrosses to the parental species or mating among F1 hybrids.

The difficulty in distinguishing different hybrid categories in this system might also be associated with the high rate of hybridization we detected. According to the criteria established here, hybrids may account for at least 40% of our total RS sample, which explains our observation of higher genetic similarity between *L. guttulus* and *L. geoffroyi* in this region, relative to allopatric populations of the same species. These results indicate that this case comprises one of the most extensive ongoing hybridization process documented for carnivores, being equivalent only to wolves and coyotes in North America [38], [39] and to wild and domestic cats in Hungary [40]. In such extensive hybrid zones, the number of possible genotypic classes to which an individual may belong increases exponentially with the number of generations over which introgression has been occurring, and therefore to distinguish among them becomes increasingly difficult, requiring a large number of loci for efficient classification [9], [41]. The extensive hybridization might also be responsible for the lack of detecting F1 hybrids in our sample. This is because in hybrid zones containing fertile hybrids (which mate among themselves and/or with parental individuals), the relative frequency of later generation admixed categories will tend to increase relative to F1 individuals, simply due to the larger number of mating combinations that produce the former.

Finally, our genetic results indicate that *L. guttulus* and *L. geoffroyi* from areas outside RS are still different genetically, supporting their current maintenance as two distinct taxonomic entities. On the other hand, their populations in RS were much more similar and, despite significant differences detected by the fixation indices and STRUCTURE analysis, individuals belonging to the two species did not form completely separated groups. This was especially the case in the central region of RS, where the proportion of admixed individuals was significantly higher than in the northern and southern areas of the state. These results highlight how the extensive introgressive hybridization process identified in this central area is promoting a substantial admixture of these two cat species at their geographic contact zone, at least with respect to neutral markers.

### Genetic, morphological and spatial characterization of RS populations

The extensive rate of hybridization detected between *L. guttulus* and *L. geoffroyi* in RS state, with the predominance of post-F1 hybrids, might indicate the absence of selection and post-zygotic barriers against the hybrids, or at least that they are limited. However, the incorporation of the morphometric analyses and the spatial assessment of genetic and morphological variation suggested the existence of some level of selection acting against hybrids, preventing the complete genetic and morphological homogenization of the two species in the regions surrounding their contact zone.

Although the first PCA results suggested an apparent lack of differentiated clusters within the RS sample, we observed that previously ignored sexual dimorphism in these species was obliterating a clear-cut pattern. Subsequent analyses conducted only with male individuals clearly indicated the occurrence of morphological heterogeneity in this region, with individuals identified as genetically pure *L. geoffroyi* presenting higher PCA scores, reflecting the overall larger size of this species relative to *L. guttulus*. We thus conclude that the apparent homogeneity observed in the first analysis was likely a consequence of the body size overlap between *L. guttulus* and female *L. geoffroyi*, more than of the hybridization process. The observed morphological segregation between the two species in RS was exactly what one would expect based on literature sources reporting their body size range in areas outside this geographic contact zone [15]–[17], [42]. Therefore, our results indicate that the usual size difference between these cat species can still be observed (and found to be statistically significant) in RS state, even in the face of extensive hybridization between them in this region.

In addition, we found that the morphological variation was significantly correlated with genetic variation and with the spatial distribution of samples, although different levels of correlation were detected (see Figure 6). The correlation between genetics and morphology was significant but moderate in intensity, because although pure *L. guttulus* individuals tended to have a smaller body size and pure *L. geoffroyi* a larger size, genetically intermediate individuals (identified as hybrids) did not necessarily show an intermediate size. In fact, the majority of male individuals identified as hybrids did not show intermediate scores in the PCA, and were clearly allocated by the DFA in their respective phenotypic population, in agreement with the *a priori* species assignments.

The observation that the DFA classified *ca.* 93% of the individuals (including hybrids) in their *a priori* phenotypic group is in stark contrast to the level of admixture estimated with the molecular data. The only two misclassified individuals (bLge46 and bLge32) did have intermediate *q* values (0.631 and 0.787, respectively), suggesting that in this case a severely admixed genome did lead to morphological misclassification. However, all other hybrid males were strongly allocated to one or the other morphological group (as much so as the pure individuals). Interestingly, in most cases this allocation did not match their highest *q* value. For the four hybrids that were morphologically allocated in *L. geoffroyi*, the *q* value assigning them to this group were 0.265, 0.483, 0.773 and 0.932, while for those morphologically assigned to *L. guttulus*, the *q* values relative to this group were even lower (0.078 and 0.191). Such discordance is not unexpected in such a complex hybrid zone, but the strength of morphological allocation to one or the other group indicates that these clusters are better defined in this area than their molecular counterparts (see below).

Cases of incongruence between morphological and genetic identification in highly hybridizing populations have also been reported in other studies [43], [44], demonstrating the serious limitation of attempting to identify hybrids *vs.* pure individuals on the basis of morphology alone. Identification of hybrids in such extensive hybrid zones using only morphological features can be challenging because introgression is often not reflected on morphology [7], [45]. Moreover, morphological introgression may follow distributions that differ from those of neutral markers, because morphological traits may be controlled by genes that are under selection, possibly related to the fitness of parental types [46]. This point is illustrated by the case of a specimen (bLge72) originally identified phenotypically as *L. geoffroyi* that presented all molecular data associated with *L. guttulus*, but clearly showed PCA scores very similar to those obtained for genetically pure *L. geoffroyi* individuals (see Figure 5C), and was also correctly associated to this phenotypic cluster in the DFA. This individual may be an example of the maintenance of one of the original phenotypes in spite of the extensive introgression of genomic segments from the other species.

Body size in this system seems to be strongly correlated with the spatial distribution of samples, with smaller individuals (more *L. guttulus*-like) strongly predominating in the region north-east of the mid-line drawn in Figure 4, and larger individuals (more *L. geoffroyi*-like) predominating south or west of this line. Even genetically identified hybrids seem to maintain the general body size associated with each geographic region, so that the correlation between spatial location and the morphological PCI is very similar with or without the inclusion of hybrids (see Results). These findings indicate that the general body size of these cats in RS may not be strongly influenced by their ongoing hybridization. Taking into account the different phytophysiognomies occurring in the north and southern parts of the state, is reasonable that different morphologies are associated with different landscape attributes favoring the smaller forms in the rugged and forested northern regions, and the larger forms in the more open regions (with less mountainous terrain) of southern RS.

Similar to our results with the morphological data, despite of the lower correlation obtained between morphologic and genetic variation, we also could detected a strong correlation between genetic and geographic distribution of the specimens evaluated, with the predominance of pure individuals on the extreme north and south of the state, and a hotspot of hybrid concentration in the central area of the state. Considering the extensive area of distribution for both species (see Figure 1A), this restricted region of hybrid concentration suggests that this hybrid zone is either quite recent (although of sufficient age to have allowed multiple generations of admixture) or stable in breadth over a long time due to the action of selection favoring the parental forms.

Stable hybrid zones can be maintained by the balance between selection and dispersal, and two different general types of selection may operate. The first one includes some sort of endogenous selection independent of the environment, represented only by intrinsic reductions on fertility or viability of the hybrids. The second one comprises exogenous selection (habitat-dependent), where different genetic combinations may be favored in different environments [1], [5], [47]. Although distinguishing between these two kinds of selection is difficult, some authors have proposed that the exogenous selection is the main force acting on stable hybrid zones [1], [5], [6], [48]. This kind of selection is generally present at the boundaries of different habitats or in environmental gradients, where the pure individuals of each parental species are adapted to specific habitats, and hybrid genotypes show higher (or sufficient) fitness within a small area of intermediate habitat. This scenario is similar to ours, in which the geographic location of the *L. guttulus* and *L. geoffroyi* hybrid zone seems to be indeed concentrated at the boundary between the types of environment mainly associated with each of the parental species. Although we currently have no information about reduction in fertility or viability of the various hybrid categories inferred to exist between these two cats, our spatial analyses indicate that the presence of habitat-dependent selection acting against hybrids is fairly plausible. Still, a conclusive answer can only be achieved with in-depth studies focusing on the ecology, physiology and behavior of these felids within and outside this contact zone.

The spatial assessment of the genetic data also revealed a pattern suggesting that the magnitude of introgression between these taxa may be asymmetric. For all of the investigated markers and analyses performed, the number of hybrid individuals morphologically resembling *L. geoffroyi* was higher than those resembling *L. guttulus*. In addition, the number of hybrids identified outside the limits of the central area defined here was larger in southern RS than towards the north. These findings indicate that genomic introgression into this population may be higher than in the opposite direction. On the other hand, it is also possible that genomic processes involving dominance and/or epistasis at morphology-related loci lead hybrids to more often resemble *L. geoffroyi* than *L. guttulus*, which might also influence their relative success and the direction of their spread.

Asymmetric introgression seems to be a common pattern in carnivore hybrid zones [49]–[54], and may be related to several aspects, such as differences in local density between the two hybridizing populations, that may lead to an increased pressure of genomic introgression in one direction *versus* the other [51]. Although very little is known about the relative densities of *L. guttulus* and *L. geoffroyi* in the wild, preliminary field data on these species indicate that the latter seems to be quite common in the vicinities of their geographic contact zone [55], indicating that uneven abundances may play a role in this process. Differences in mating system and physiological characteristics of each species, including different estrus periods, parental care and socialization may also favor asymmetric pressures of introgression [49], [54], [56]. However, given the scarcity of information on any of these aspects in these cats, it is presently difficult to evaluate whether they may influence this apparent asymmetry in introgression. Different selective pressures against foreign alleles may also be acting in each species, allowing more genes to pass in one direction than the other [47], [57]. In this case, different selective pressures may act on the two sides of this hybrid zone, with selection favoring hybrids that mate with *L. geoffroyi*-like individuals, possibly exhibiting a lower reduction in viability and fertility.

In conclusion, this study demonstrated that the rate of hybridization and introgression between *L. guttulus* and *L. geoffroyi* in RS state is quite high, with our sample being composed of a large proportion of post-F1 hybrids. In spite of the high rates of introgression, which should lead to rapid homogenization between the two cat species around their geographical contact zone, our morphological and spatial evaluations indicate that they remain significantly distinct. In this context, we consider it likely that different selective pressures (possibly related to body size) play a role in maintaining this morphological distinctiveness, as well as in restricting the geographic breadth of the hybrid zone to the vicinities of the contact region between the two species. Investigating this hypothesis with integrated genomic and ecological approaches should shed light onto the historical and current processes influencing the dynamics of this hybrid zone, and open up new avenues for future research focusing on this complex system.

## Supporting Information

Figure S1 **Species specific-haplotypes described by ref. [13] depicted as haplotype networks.** (A) mtDNA *ND5* gene, (B) X-linked introns of genes *PLP1* and *BTK*, (C) Y-linked introns of genes *ZFY* and *SMCY3*. Each unique haplotype is represented by a circle whose size is proportional to its frequency. Colors indicate the frequency of the haplotype in each population group: dark grey for *Leopardus guttulus*, light grey for *Leopardus geoffroyi*, and white for *L. colocolo*. Each haplotype nomination was the same used in [13], and the absence here of some haplotypes described in that study is due to the exclusion of the samples assigned to *L. tigrinus* from Northeastern Brazil.
(TIF)

Table S1 **Samples analyzed in the present study.** The parental populations of both species include only the individuals used in the genotypes simulation.
(DOCX)

Table S2 **Haplotypes used in this study from reference [13] and their respective GenBank accession numbers.**
(DOCX)
