# Supplementary figures and images for: Comparative Assessment of Genetic and Morphological Variation at an Extensive Hybrid Zone between Two Wild Cats in Southern Brazil
Source: PLoS One. 2014 Sep 24;9(9):e108469. doi: 10.1371/journal.pone.0108469 (PMC4177223; doi:10.1371/journal.pone.0108469)

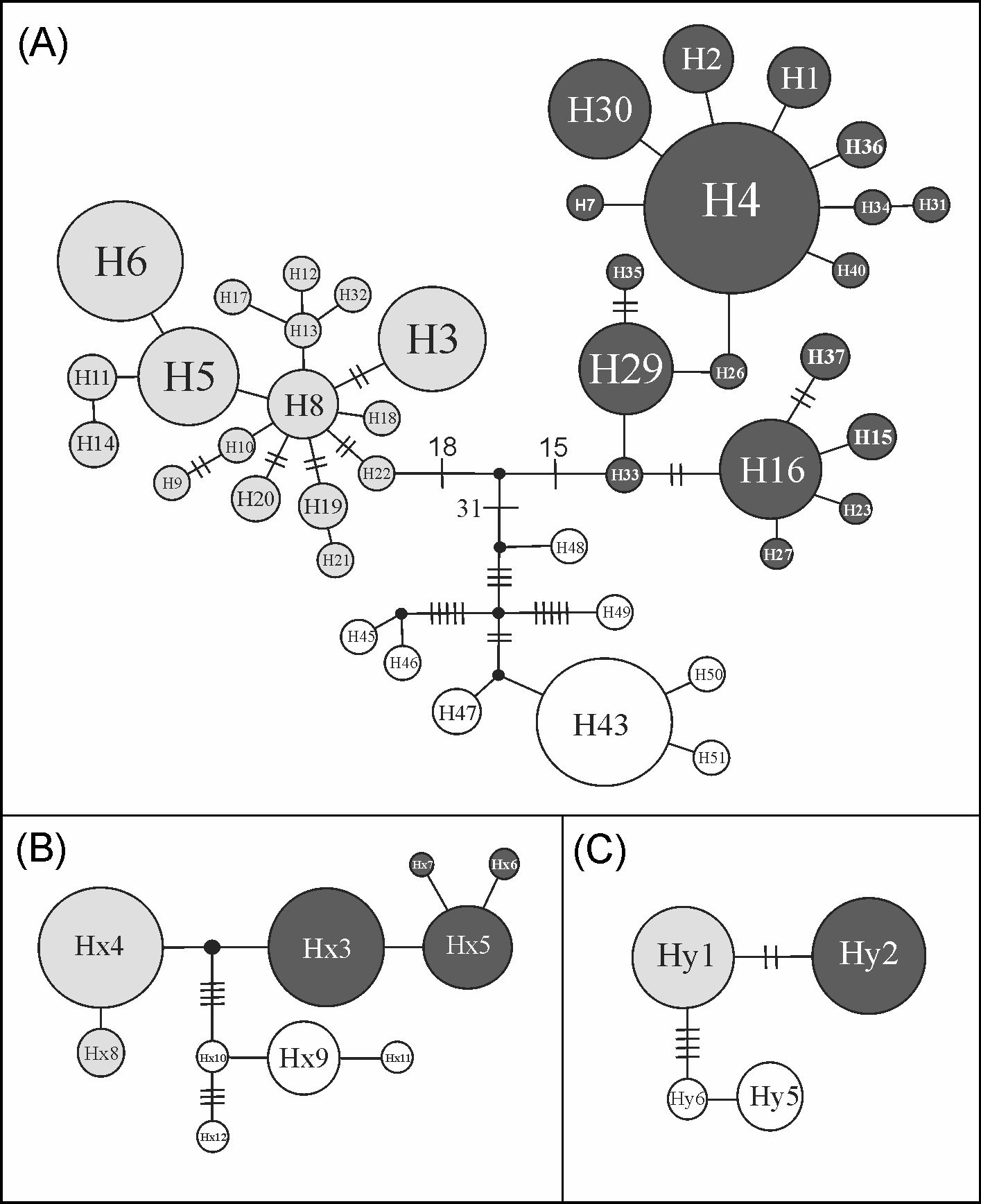

Supplement: Figure S1 — Species specific-haplotypes described by ref. [13] depicted as haplotype networks. (A) mtDNA ND5 gene, (B) X-linked introns of genes PLP1 and BTK, (C) Y-linked introns of genes ZFY and SMCY3. Each unique haplotype is represented by a circle whose size is proportional to its frequency. Colors indicate the frequency of the haplotype in each population group: dark grey for Leopardus guttulus, light grey for Leopardus geoffroyi, and white for L. colocolo. Each haplotype nomination was the same used in [13], and the absence here of some haplotypes described in that study is due to the exclusion of the samples assigned to L. tigrinus from Northeastern Brazil. (TIF) [file pone.0108469.s001.tif]
