# Supplementary material for: Comparative Assessment of Genetic and Morphological Variation at an Extensive Hybrid Zone between Two Wild Cats in Southern Brazil
Source: PLoS One. 2014 Sep 24;9(9):e108469. doi: 10.1371/journal.pone.0108469 (PMC4177223; doi:10.1371/journal.pone.0108469)
Supplement: Table S1 — Samples analyzed in the present study. The parental populations of both species include only the individuals used in the genotypes simulation. (DOCX) [file pone.0108469.s002.docx]

Table S1

| **Samples** | **Gender** | **Location (geographic origin)** | **Institution/contact** | |
| --- | --- | --- | --- | --- |
| ***Leopardus guttulus*  Rio Grande do Sul (n = 45)** | | | |  |
| bLgut01 | F | Triunfo | E. Eizirik | |
| bLgut04 | ? | Rio Grande do Sul | Zoo Sapucaia do Sul | |
| bLgut05 | M | Rio Grande do Sul | Zoo Sapucaia do Sul | |
| bLgut06 | ? | Cachoeira do Sul | E. Eizirik | |
| bLgut09 | F | Cachoeira do Sul | Zoo Cachoeira do Sul - E. Salomão | |
| bLgut10 | F | Guaporé | Zoo Sapucaia do Sul | |
| bLgut46 | M | Garibaldi | ZooParticular Maison Forestier, Seagram | |
| bLgut47 | F | Garibaldi | ZooParticular MaisonForestier, Seagram | |
| bLgut48 | F | Estrela | Zoo Sapucaia do Sul | |
| bLgut49 | F | Guaíba | P. Ott | |
| bLgut51 | M | Santa Cruz do Sul | D. Sana | |
| bLgut68 | M | Montenegro | L. F. Brutto | |
| bLgut69* | M | Santa Cruz do Sul | T. Breier | |
| bLgut79* | F | Eldorado do Sul | T. R.O. Freitas, J. da Silva, F. Bitencourt | |
| bLgut80* | M | Glorinha | J. Mähler Jr., C. B. Indrusiak | |
| bLgut94 | M | Ibarama | S. Cechin, K. H. Toscan, L. O. M. Giasson | |
| bLgut95 | ? | Sarandi | J. Mähler Jr. | |
| bLgut98 | F | Restinga Seca | J. Mähler Jr. | |
| bLgut99 | M | Nova Esperança do Sul | P. R. Vielmo | |
| bLgut100 | M | Santo Antônio da Patrulha | V. Andrade, J. da Silva | |
| bLgut102* | F | Erechim | Zoo Sapucaia do Sul | |
| bLgut106* | M | Santa Cruz do Sul | Arca de Noé, Criadouro Conservacionista de Morro Reuter | |
| bLgut108 | M | Santa Maria | Arca de Noé, Criadouro Conservacionista de Morro Reuter | |
| bLgut110 | M | Itapuã | M. F. Corrêa | |
| bLgut113 | M | Getúlio Vargas | IBAMA RS | |
| bLgut117 | F | Arroio do Meio | C. B. Kasper | |
| bLgut119* | M | Cachoeira do Sul | Zoo Cachoeira do Sul / E. Salomão | |
| bLgut120* | M | Cachoeira do Sul | Zoo Cachoeira do Sul / E. Salomão | |
| bLgut121* | M | Triunfo | Zoo Sapucaia do Sul | |
| bLgut122* | M | Arroio do Sal | C. E. Rovedder, M. Repenning, T. S. da Silveira | |
| bLgut124* | M | Arroio do Meio | E. Pedó | |
| bLgut131 | M | Guaporé | L. Pinto | |
| bLgut132 | M | Rolante | J. Marinho | |
| bLgut133 | M | Lagoa Vermelha | R. Schmidt | |
| bLgut134 | F | Dom Pedro de Alcântara | I. P. Coelho | |
| bLgut135* | F | Estância Velha | Zoo Sapucaia do Sul | |
| bLgut136* | M | Carazinho | Zoo Universidade Passo Fundo | |
| bLgut137* | M | Morro Reuter | I. Rollet | |
| bLgut138* | F | Machadinho | J. A. da Rosa | |
| bLgut139* | M | Rio Grande do Sul | MCN3062 - FZBRS | |
| bLgut140* | M | Ibarama | MCN3056 – FZBRS | |
| bLgut141 | ? | Arroio do Sal | J. Mähler Jr. | |
| bLgut142 | M | Rondinha | M. B. Martins, G. Vinciprova | |
| bLgut143* | F | Novo Hamburgo | SMAM Novo Hamburgo | |
| bLgut146 | F | Cachoeira do Sul | A. Senra | |
| bLgut149* | M | Forquetinha | E. Pedó | |
|  |  |  |  | |
| **Parental *L. guttulus* (n = 32)** | | | |  |
| bLgut30 | F | Paraguai | Refugio Itaipu Paraguaio | |
| bLgut53 | M | Valença, Rio de Janeiro | Zoo Rio de Janeiro | |
| bLgut54 | F | Mogi Guaçu, São Paulo | Zoo de Mogi Guaçu | |
| bLgut55 | M | Limeira, São Paulo | Zoo Limeira | |
| bLgut56 | M | Piracicaba, São Paulo | Zoo Piracicaba | |
| bLgut58 | M | Americana, São Paulo | Zoo São Bernardo do Campo | |
| bLgut59 | F | Mogi Guaçú, São Paulo | Zoo São Bernardo do Campo | |
| bLgut60 | M | Rafard, São Paulo |  | |
| bLgut61 | M | São Carlos, São Paulo |  | |
| bLgut62 | M | Campinas, São Paulo |  | |
| bLgut64 | M | São José do Rio Pardo, São Paulo | Zoo São José do Rio Pardo | |
| bLgut66 | F | Sorocaba, São Paulo | Zoo São José do Rio Pardo | |
| bLgut70 | M | Sorocaba, São Paulo | Zoo Sorocaba | |
| bLgut71 | M | Sorocaba, São Paulo | Zoo Sorocaba | |
| bLgut72 | M | Miranda, Mato Grosso do Sul |  | |
| bLgut73 | M | Campinas, São Paulo |  | |
| bLgut75 | F | Bauru, São Paulo |  | |
| bLgut76 | M | Pedreira, São Paulo |  | |
| bLgut78 | M | Leme, São Paulo |  | |
| bLgut84 | ? | Jundiaí, São Paulo |  | |
| bLgut87 | M | Mogi Mirim, São Paulo |  | |
| bLgut89 | M | Pato Branco, Paraná | Zoo Cascavel | |
| bLgut93 | M | Curitiba, Paraná | Zoo Curitiba | |
| bLgut96 | M | Água Boa, Mato Grosso | F. Rodrigues | |
| bLgut97 | M | Domingos Martins, Espírito Santo | CENAP | |
| bLgut103 | F | Jundiaí, São Paulo | CENAP | |
| bLgut109 | M | Serra da Canastra, Minas Gerais | F. Rodrigues | |
| bLgut114 | M | Ilha de São Francisco do Sul, Santa Catarina |  | |
| bLgut123* | M | Bom Retiro, Santa Catarina | C. Castilho, L. G. M. de Sá | |
| bLgut125 | M | Santa Catarina |  | |
| bLgut127 | M | Santa Catarina |  | |
| bLgut220 | M | Celso Ramos, Santa Catarina | A. Costa | |
|  |  |  |  | |
| ***Leopardus geoffroyi* Rio Grande do Sul (n = 49)** | | | |  |
| bLge01 | M | Santa Cruz do Sul | Zoo Sapucaia do Sul | |
| bLge02 | M | Cachoeira do Sul | Zoo Cachoeira do Sul | |
| bLge03 | ? | Cachoeira do Sul | Zoo Cachoeira do Sul | |
| bLge04 | M | Cachoeira do Sul | Zoo Cachoeira do Sul | |
| bLge05 | M | Cachoeira do Sul | Zoo Cachoeira do Sul | |
| bLge06 | ? | Cachoeira do Sul | Zoo Cachoeira do Sul | |
| bLge07 | M | Cachoeira do Sul | Zoo Cachoeira do Sul | |
| bLge08 | M | Caçapava do Sul | D. Sana | |
| bLge10 | F | Cachoeira do Sul | Zoo Cachoeira do Sul | |
| bLge11 | M | Pantano Grande | E. Salomão | |
| bLge12 | M | Cachoeira do Sul | E. Salomão | |
| bLge13 | M | Eldorado do Sul | P. Ott | |
| bLge28* | M | Camaquã | L. Veronese | |
| bLge29* | M | Quaraí | T. C. Trigo | |
| bLge31* | M | Quaraí | T. C. Trigo | |
| bLge32* | M | Pantano Grande | T. C. Trigo | |
| bLge33* | M | Alegrete | M. B. Martins | |
| bLge35 | M | Rio Grande | T. C. Trigo | |
| bLge36 | F | Rio Grande | T. C. Trigo | |
| bLge37* | F | São Lourenço do Sul | Zoo Sapucaia do Sul | |
| bLge38 | F | Santa Maria | L. Cabral | |
| bLge39 | M | Jaguari | R. Zachia | |
| bLge41 | M | Itaqui | E. R. Behr, L. O. M. Giasson | |
| bLge42 | M | Barra do Ribeiro | C. B. Indrusiak | |
| bLge43 | M | São Borja | C. B. Indrusiak | |
| bLge44 | F | São Gabriel | F. Michalski | |
| bLge46* | M | Canela | Zoo Sapucaia do Sul | |
| bLge47* | M | São Leopoldo | Zoo Sapucaia do Sul | |
| bLge49* | M | Rio Grande do Sul | Zoo Sapucaia do Sul | |
| bLge70 | F | Arroio Grande | Zoo Sapucaia do Sul | |
| bLge71* | F | Rio Grande | T. R. O. de Freitas, J. Stoltz | |
| bLge72* | M | Encruzilhada do Sul | Quinta da Estância Grande | |
| bLge73* | M | Cachoeira do Sul | Zoo Cachoeira do Sul | |
| bLge74* | F | Piratini | F. D. Mazim | |
| bLge75* | F | Arroio Grande | F. D. Mazim, J. B. G. Soares | |
| bLge76* | M | Arroio Grande | F. D. Mazim, J. B. G. Soares | |
| bLge77* | M | Santana do Livramento | F. D. Mazim | |
| bLge78* | F | Rio Grande | F. D. Mazim | |
| bLge79 | M | São Lourenço do Sul | CETAS UFPel | |
| bLge80 | M | São Lourenço do Sul | CETAS UFPel | |
| bLge89 | M | Alegrete | T. R. O. de Freitas, J. Marinho | |
| bLge90 | M | São Gabriel | F. Michalski | |
| bLge91* | M | Itaqui | FZBRS | |
| bLge92* | M | Alegrete | FZBRS | |
| bLge93* | F | Arroio Grande | F. D. Mazim | |
| bLge94* | F | Pelotas | F. D. Mazim | |
| bLge95* | F | Cristal | G. A. Bencke | |
| bLge96* | F | Pelotas | F. D. Mazim | |
| bLge97 | F | Guaíba | E. Borsato | |
|  | | | |  |
| **Parental *L. geoffroyi* (n = 20)** | | | |  |
| bLge09 | F | Córdoba, Argentina | Zoo Cordoba | |
| bLge20 | M | Uruguai | Museo de Ciencias Naturales | |
| bLge51 | M | Província de Buenos Aires, Argentina | Parque Zoológico de La Plata | |
| bLge52 | M | Catamarca, Argentina | Parque Zoológico de Córdoba | |
| bLge54 | M | Montevidéu, Uruguai | Parque Zoológico Mercedes | |
| bLge55 | F | Uruguai | Centro de Reprodução Cerro Pan de Azucar | |
| bLge56 | M | Uruguai | Centro de Reprodução Cerro Pan de Azucar | |
| bLge57 | F | Uruguai | Centro de Reprodução Cerro Pan de Azucar | |
| bLge62 | F | Santa Cruz Dept., Bolívia | Santa Cruz Zoo | |
| bLge63 | M | Santa Cruz Dept., Bolívia | Santa Cruz Zoo | |
| bLge64 | M | Santa Cruz Dept., Bolívia | Santa Cruz Zoo | |
| bLge65 | F | Beni Dept., Bolívia | Santa Cruz Zoo | |
| bLge66 | M | Beni Dept., Bolívia | Santa Cruz Zoo | |
| bLge68 | F | Cochabamba Dept., Bolívia | Santa Cruz Zoo | |
| bLge81 | ? | Bolívia |  | |
| bLge82 | M | Bolívia |  | |
| bLge84 | ? | Bolívia |  | |
| bLge85 | M | Bolívia |  | |
| bLge86 | M | Bolívia |  | |
| bLge87 | M | Bolívia |  | |

* Specimens included in morphological analyses.

M = male, F = female.
