# Supplementary material for: Comparative Assessment of Genetic and Morphological Variation at an Extensive Hybrid Zone between Two Wild Cats in Southern Brazil
Source: PLoS One. 2014 Sep 24;9(9):e108469. doi: 10.1371/journal.pone.0108469 (PMC4177223; doi:10.1371/journal.pone.0108469)
Supplement: Table S2 — Haplotypes used in this study from reference [13] and their respective GenBank accession numbers. (DOCX) [file pone.0108469.s003.docx]

Table S2

| Haplotype | GenBank accession number |
| --- | --- |
| Mitochondrial DNA |  |
| H1 | KF679912 |
| H2 | KF679913 |
| H3 | KF679914 |
| H4 | KF679915 |
| H5 | KF679916 |
| H6 | KF679917 |
| H7 | KF679918 |
| H8 | KF679919 |
| H9 | KF679920 |
| H10 | KF679921 |
| H11 | KF679922 |
| H12 | KF679923 |
| H13 | KF679924 |
| H14 | KF679925 |
| H15 | KF679926 |
| H16 | KF679927 |
| H17 | KF679928 |
| H18 | KF679929 |
| H19 | KF679930 |
| H20 | KF679931 |
| H21 | KF679932 |
| H22 | KF679933 |
| H23 | KF679934 |
| H26 | KF679937 |
| H27 | KF679938 |
| H29 | KF679940 |
| H30 | KF679941 |
| H31 | KF679942 |
| H32 | KF679943 |
| H33 | KF679944 |
| H34 | KF679945 |
| H35 | KF679946 |
| H36 | KF679946 |
| H37 | KF679948 |
| H40 | KF679951 |
| H43 | KF679954 |
| H45 | KF679956 |
| H46 | KF679957 |
| H47 | KF679958 |
| H48 | KF679959 |
| H49 | KF679960 |
| H50 | KF679961 |
| H51 | KF679962 |
| X-chromosome |  |
| Hx3 | KF679967/KF679905 |
| Hx4 | KF679968/KF679908 |
| Hx5 | KF679969/KF679905 |
| Hx6 | KF679969/KF679907 |
| Hx7 | KF679969/KF679906 |
| Hx8 | KF679970/KF679908 |
| Hx9 | KF679971/KF679909 |
| Hx10 | KF679972/KF679909 |
| Hx11 | KF679973/KF679909 |
| Hx12 | KF679974/KF679910 |
| Y-chromosome |  |
| Hy1 | KF679981/KF679976 |
| Hy2 | KF679982/KF679977 |
| Hy5 | KF679983/KF679979 |
| Hy6 | KF679983/KF679980 |
